# Programmatic Impact of Implementing GeneXpert MTB/ RIF Assay for the Detection of *Mycobacterium Tuberculosis* in Respiratory Specimens from Pulmonary Tuberculosis Suspected Patients in Resource Limited Laboratory Settings of Eastern Nepal

**DOI:** 10.2174/1874285801812010009

**Published:** 2018-02-28

**Authors:** Prakash Shrestha, Hemanta Khanal, Prasanna Dahal, Pranita Dongol

**Affiliations:** 1 Department of microbiology, Sunsari Technical College, Dharan-4, Sunsari, Nepal; 2 Central Campus of Technology, Tribhuvan University, Hattisar, Dharan, Sunsari, Nepal; 3 Department of Pharmacy, Tribhuvan University, Dharan-4, Sunsari, Nepal

**Keywords:** Mycobacterium tuberculosis, Utility, Impacts, GeneXpert MTB/RIF assay (Xpert assay), Multidrug resistance Tuberculosis (MDR), Rifampin resistance

## Abstract

**Background:**

In Nepal, introduction of GeneXpert MTB/RIF assay (Xpert assay) as an initial confirmation test for tuberculosis (TB) has been considered to have impact as a significant decrease in number of clinically diagnosed pulmonary tuberculosis (PTB) cases than previous years. This study aims to find out the distribution profile of suspected tuberculosis cases according to patients age, gender, treatment history and HIV status as well as to evaluate the utility of the Xpert assay over conventional acid-fast bacilli (AFB) staining method for the proper diagnosis of *M. Tuberculosis* in respiratory specimens from the tuberculosis (TB) suspected patient samples.

**Methods:**

The prospective cross-sectional analytical study was conducted in National Anti-Tuberculosis Center (NATA) center- Biratnagar and Primary Healthcare Center (PHC) - Manglabare, Morang District, of eastern Nepal from January 2014 to August 2014. Laboratory investigation was done by conventional AFB staining followed by Xpert assay.

**Results:**

A total of 1549 sputum samples were initially analyzed. AFB staining resulted in 1441 AFB smear negative samples and 88 AFB smear positive samples, whereas 20 samples were directly processed for Xpert assay. The male: female smear positive ratio was 2.8:1 and was higher among age groups (21-40) years. Tuberculosis among HIV patients was found 22.22%. Xpert assay demonstrates that out of 1441 smear negative AFB cases, 258 were found to have TB positive, whereas out of 88 smears positive AFB cases 12 were found to have TB negative. The sensitivity of the Xpert assay in patients classified as AFB smear positive was found 85.4% and the specificity in smear negative patients was 81%.

**Conclusion:**

The study concluded that implementation of Gene Xpert MTB/RIF assay is a helpful tool for early and rapid detection of tuberculosis with greater sensitivity and specificity over traditional AFB staining techniques.

## 1 INTRODUCTION

*Mycobacterium Tuberculosis* have affected human health for at least 70,000 years, yet whose immunological workings remain scarce [1, 2]. World Health Organization (WHO) has estimated that around 9.0 million people globally, equivalent to 126 cases per 100,000 populations are infected with disease [3]. It is also estimated that in Nepal the prevalence of all types of tuberculosis (TB) cases is 66,000 (241/100K) while the number of all forms of incidence cases is around 45,000 (163/100K) [4].

The Cepheid GeneXpert MTB/RIF assay (Xpert assay) is an automated and a real-time Polymerase Chain Reaction (PCR) based test offering rapid identification of *M. Tuberculosis* and detection of Rifampicin resistance directly from clinical samples within 2 hours [5]. The fully integrated and automated equipment is composed of a personal computer, barcode scanner and preloaded software; single-use disposable cartridges containing lyophilized reagents,buffers and washes. A six-color laser detecting device is employed for target detection and characterization. This technique utterly simplifies the three processes *i.e*. specimen preparation, amplification and detection required for real-time PCR-based molecular testing [6]. Besides sputum sample, this system is also appraised in TB diagnosis and identifying Rifampicin susceptible organisms from samples like cerebrospinal fluid, gastric aspirate, pleural fluid, tissues and pus/fluid from lymph nodes [7-9].

Approximately 95% of *M. Tuberculosis* isolates have been reported as Rifampicin resistant serving as a marker for multidrug resistant tuberculosis (MDR-TB) [10-12]. The Xpert assay utilizes five molecular beacons that detect mutations in 81-bp “core” region of the *rpoB* gene associated with Rifampicin resistance [13]. WHO recommends using the Xpert assay as a primary diagnostic test for persons suspected with drug resistant tuberculosis (DR-TB) or in settings with high HIV prevalence and as a second test for those with an abnormal chest x-ray (CXR) or negative smear microscopy results [14-16]. The use of Xpert assay started in Nepal since 2012 and mainly three organizations held the capacity of this technology: International Organization for Migration (IOM), Health Research and Social Development Forum (HERD) and National Tuberculosis Center (NTC) [17]. According to report of tuberculosis in the Southeast Asian region, in Nepal there has been a significant decrease in numbers of clinically diagnosed pulmonary tuberculosis (PTB) cases than previous years, this decrease is known to be greatly related to reduction of misdiagnosis due to the introduction of Xpert assay as an initial confirmation test for TB [4]. Globally, there has been prominent utilization of the Xpert assay test across a variety of settings, with over 2.3 million cartridges procured in the public sector. The Xpert assay for is found to be more consistent for detection of *M. tuberculosis* and rifampicin resistant in patients with either significant clinical indications of tuberculosis or smear negative sputum’s [18, 19]. Many researches are conducted all over the world to evaluate the performance of Xpert assay against culture, acid-fast stain and fluorescent microscopy of *M*. *Tuberculosis* and the findings had shown the Xpert assay is a novel invention [19-22]. This study aims to provide the distribution profile of suspected tuberculosis cases, according to patients age, gender, treatment history and HIV status as well as to evaluate the effectiveness of Xpert assay over conventional AFB staining method for the proper diagnosis of *M. Tuberculosis* in respiratory specimens from the patients along with detection of rifampicin drug resistance in resource limited tuberculosis treatment centers of eastern Nepal. The main reasons for identifying rifampicin resistance through Xpert assay in this study is due to its speedy test result and availability of rifampicin resistance test specific Xpert cartridge only in the study centers. Normally to get any drug resistance results takes weeks.

## 2 MATERIALS AND METHODS

Prospective cross-sectional, analytical study was undertaken in National Anti-Tuberculosis Association (NATA) Directly Observed Treatment Short course (DOTS) Plus center, Biratnagar, and Primary Health Care center (PHC)-Manglabare of Morang district, Nepal for the period of 8 months from January to August 2014. The pulmonary samples (sputum) collected from the suspected individuals, new smear negative patients with high suspension of tuberculosis, retreatment cases, people living with HIV (PLHIV) suspected TB patients and patients who still remain smear positive for 3 months of Category I and Category II anti-tubercular treatment. Sputum collection, processing, microscopic examination of AFB and Xpert assay for TB and MDR-TB were performed at NATA hospital, Biratnagar and PHC-Manglabare center. Tuberculosis other than pulmonary tuberculosis was excluded from the study. Institutional permission was obtained from the study centers. Ethical approval was not required to carry out this work as the respiratory specimens were collected as part of the routine patient care investigation in the hospital and collected clinical samples were provided by the study centers. Data were obtained from the patients visiting NATA DOTS plus center and PHC-Manglabare through routinely maintained TB Xpert assay log book provided by Nepal IOM TB Reach project. The data included, socio-demographic variable such as name of patients, gender, age, district, residence, etc. and other variable their HIV status, smear results and Xpert assay results.

### 2.1 Specimen Collection and Processing

Each patient was provided with two sterile vials for collection of sputum samples: one for early morning representing overnight secretions and another for spontaneously expectorated on the spot. The samples with saliva or obvious food particles or other solid particulate were rejected and requested again. The spot sputum sample was discarded if the morning sample was proper and adequate. The morning unprocessed sputum sample (4mL) was mixed with 8mL of sample reagent and was concentrated by centrifugation. The N-acetyl-L-cysteine–NaOH (NALC-NaOH) method was used to digest, decontaminate, and concentrate respiratory specimens and was stored at 2-8^0^C in case of delayed diagnostic procedure [23].

### 2.2 AFB Smears

After processing of the specimens, smears were prepared from all sputum samples and were examined at NATA DOTS plus center by fluorescence staining methods and by the Ziehl-Neelsen (ZN) staining method at PHC- Manglabare for the presence of AFB and examined by fluorescence microscope and light microscope respectively.

### 2.3 Xpert Assay

All direct 1mL sputum samples were processed for Xpert assay per the manufacturer’s instructions (Cepheid, Sunnyvale, CA, USA). For processed sputum pellets, 0.5ml was treated with 1mL sample reagents, mixed well, incubated for 15 min at room temperature, and then the entire content of 1.5ml was transferred to the Xpert cartridge. Cartridges were inserted into the Xpert assay device, and the automatically generated results were read after 1 hour 50 minutes.

The Xpert assay was done directly after a smear test if CXR is not available. For people living with HIV (PLHIV), MDR suspect microscopy and Xpert assay were performed at the same time.

### 2.4 Statistical Analysis

Data reading was done after the completion of Xpert assay and coded to simplify the process of data entry. The data were entered in MS Excel 2007 and the database was created in SPSS 16.0 for Windows. After correcting the error, the data was being transferred to variables as required and analyzed. Summary output tables of descriptive statistics cross tabulation between various variables were produced. In association of factors Chi- square test was used. The test was considered statistically significant if the p-value obtained was less than 0.05. The sensitivity and specificity of each Xpert assay test were calculated according to following formula.

$\mathrm{Sensitivity}\;=\;\frac{\mathrm{Tru}\;\mathrm{positive}\;(\mathrm{TP})}{\mathrm{True}\;\mathrm{positice}\;(\mathrm{TP})+\mathrm{False}\;\mathrm{positive}\;(\mathrm{FP})}\times 100\%$

$\mathrm{Speficity}\;=\;\frac{\mathrm{Tru}\;\mathrm{negative}\;(\mathrm{TN})}{\mathrm{True}\;\mathrm{negative}\;(\mathrm{TN})+\mathrm{False}\;\mathrm{positive}\;(\mathrm{FP})}\times 100\%$

## 3 RESULTS

In this study, 1549 patient’s sputum samples were collected and analyzed. Among the total study samples 995 (64.23%) were taken from NATA DOTS plus center, Biratnagar and 554 (35.77%) were obtained from PHC-Manglabare.

### 3.1 Distribution of Suspected Cases and Xpert Assay Result Among Gender and Age Groups

Among the total sample population from both health facilities, the proportion of males was higher than females, *i.e*. 992 (64.04%) males vs 557 (35.96%) females. Higher numbers of patients visited NATA DOTS plus center, Biratnagar as it is located in a metropolitan city. Among 992 suspected male cases who had the Xpert assay in NATA DOTS plus center and PHC-Manglabare, 223 were TB positive, 692 were TB negative and 57 tests were failed (*i.e*. invalid, error and without result cases). Distribution profile of suspected cases among male and females and Xpert assay results for TB positive, negative and failed cases is shown in Table (**1**) Furthermore, in this study, we found that highest occurrence of TB among population by age group between 21-40 years, followed by 41-60 and 61-80 age groups (Table **2**).

### 3.2 Distribution of Suspected Cases and Xpert Assay Result, According to Treatment History

In this study, Xpert assay was performed on 1455 samples of new suspects and 94 samples of previously treated cases. The Xpert assay found that 268 were infected with TB among new cases, whereas 1103 were negative and 84 tests were failed. Among the positive cases 234 had rifampicin sensitivity, 10 were rifampicin resistance and 24 were TB positive but rifampicin indeterminate. Among previously treated cases, 63 were infected with TB, 24 were negative and seven tests were failed. Distribution of TB positive and negative cases among new and previously treated cases is shown in Table (**3**).

### 3.3 Distribution of Suspected Cases and Xpert Assay Result, According to the HIV Status

In our study, distribution of suspected sample cases and the Xpert assay result, according to the HIV status shows that among the total suspected cases only nine were HIV positive and remaining 984 were HIV negative Table (**4**) and 25% of HIV positive cases were TB positive.

### 3.4 Distribution of Xpert Assay Result Among MDR Suspected Cases and MDR Not Suspected Cases

In this study, out of total samples 126 were suspected as multidrug resistant (MDR) tuberculosis. MDR suspicion was done on the basis of smear result and clinical history. Among MDR suspected cases 84 (66.7%) were TB positive, whereas 247 (17.3%) were positive among non suspected cases. Among the positive cases, 216 were rifampicin sensitive, nine were rifampicin resistance and 22 were TB positive, but rifampicin indeterminate as shown in Fig. (**1**).

The data were statistically significant (p-value < 0.005.)

### 3.5 Distribution of Suspected Cases With the Xpert Assay Result Over AFB Smear Result

Among 1549 sputum samples, AFB staining resulted in 1441 AFB smear negative samples and 88 AFB smear positive samples, whereas 20 samples were directly processed for Xpert assay. Xpert assay of 1441 smear negative AFB cases shows 258 were TB positive, among which 222 had rifampicin sensitivity, 10 were rifampicin resistance, 26 were TB positive but rifampicin indeterminate. Out of 88 smears positive AFB cases, 70 were TB positive with 57 Rifampicin sensitivity, 12 rifampicin resistance, 1 was TB positive but rifampicin indeterminate cases and among the 20 samples which were directly tests for Xpert assay results, three were TB positive with rifampicin sensitivity as shown in Table (**5**).

### 3.6 Sensitivity and Specificity in detecting M. *Tuberculosis* by Xpert Assay Compared to AFB Results

The sensitivity of the Xpert assay in patients classified as AFB smear positive was found to be 85.4% (70/82), the specificity of AFB smear negative patients was 81% (1099/157) as shown in Table (**6**)**.** All the value was calculated in 95% CI by Medicalc^®^.

## 4 DISCUSSION

The incidence of pulmonary TB in our study was higher in male than female. Our study shows that male: female ratio of positive cases is 2.8:1 which is slightly higher than 2.2:1 shown by Nepal tuberculosis Program, Annual report 2013 [24]. Furthermore, in this study, we found that highest occurrence of TB among population in age group between 21-40 years, followed by 41-60 and 61-80 age groups. This result agrees with the findings obtained in the study conducted by US Government “Tuberculosis in the United States: National Tuberculosis Surveillance System”, in the manner that TB is primarily a disease that affects young adults in their most productive years. In the United States in 2006, 63% of the TB cases were among those ages 25 – 64 [25]. Previous treatment for TB is the most important risk factor for development of MDR-TB. In our study, 18.5% of new cases and 67% of previously treated cases developed TB. This study shows 10 (0.68%) drug resistant cases were among new cases and 12 (12.7%) resistant cases were among the treated cases. In 2006, estimates of drug resistant tuberculosis (DR-TB) prevalence was 2.9% among new cases and 11.7% among previously treated cases which is comparable to our findings in this study [24, 26]. Similarly, Drug Resistance Survey conducted by GENETUP in collaboration with WHO in 2011 also produced estimates 2.2% among new cases and 15.2% among previously treated cases of MDR-TB [24].

Among the total TB cases 0.6% cases were HIV positive, which is comparable to the surveillance done in 1995/1996 but contrast based on the data of sentinel survey, 2011/12 which shows that co-infection rate in Nepal is 2.4% (HIV among TB) and 11.6% (TB among HIV). Similarly, Highest HIV-TB co-infection (64.88%) was reported in 2003 but was only 5.97% in 2012 [27, 28].

Furthermore, this study shows that there is 17.9% positivity among the smear negative cases, 79.55% positivity among smear positive cases as compared with that of AFB results. This result is comparable to results presented in the Nepal IOM TB Reach assay test result 2014 which shows that 17% positivity among smear negative cases, 85% positivity among smear positive cases (slightly higher than our result)and 38% positivity among direct Xpert assay cases (half more than our result). 5.89% tests were failing in our result which is lower than Nepal IOM TB Reach MTB/Rif test result 2014 which is 9.5% [29]. Most of the tests failed due to problem in power supply and other problem may be cartridge problem and lack of good samples. Among the 1458 valid tests 18.5% cases are MDR-TB and 22.7% were positivity cases. Among overall positive cases 6.64% cases are MDR-TB. In a similar study carried out by Creswell et al in nine Xpert assay centers of Nepal for detecting outcome of the Xpert assay showed that positivity was found to be 15.9% among positive cases, 7.6% were Rifampicin resistant and 10.7% tests were failed [17]. In another study conducted by Iram et al to evaluate the diagnostic accuracy of the Xpert assay in pulmonary and extra-pulmonary tuberculosis cases in comparison with conventional techniques show that Xpert assay diagnose an additional 23.4% case positive over ZN staining along with an added advantage of much lower turnaround time suggesting Xpert assay is a sensitive method for rapid diagnosis of tuberculosis, especially in smear negative cases [30].

The sensitivity of the Xpert assay in patients classified as AFB smear positive was found to be 85.4% (70/82), the specificity of AFB smear negative patients was 81% (1099/157), which was higher than that found by Park et al., in similar study [31], however, was lower than that conducted by Ioannidis et al were the sensitivity, specificity, as well as positive and negative predictive values were 90.6%, 94.3%, 93.5%, and 91.7% respectively in pulmonary samples [18]. In our study, the NPV was 98.9% (1099/1111) and the positive predictive value, PPV was 21.3% (70/328). Similarly positive likelihood ratio and negative likelihood ratio are 4.49 and 0.2. Pinyopornpanish et al in similar studies in northern Thailand found that, the sensitivity (SEN) and specificity (SPEC) for Xpert results were 95.3% and 86.4%, while using MGIT sputum culture as a reference standard and concluded that Xpert assay have good sensitivity and specificity of in detecting *M. Tuberculosis* from sputum specimens, especially among patients who had acid-fast negative sputum smear [32].

## 5 LIMITATION AND RECOMMENDATIONS

The Xpert assay has certain limitations in this study. Majority (60%) of the failed cases is due to the power cut off, so provisions for continuous power supply should be made. However, the need for retraining and supervision cannot be overlooked as staff turnover is a constant reality. As a part of training, ensuring solidified National testing and treatment guidelines are in place is critical. Country adoption of the WHO policy guidance around testing algorithms as well as recent WHO new case and outcome definitions incorporating Xpert assay and other WHO recommended tests, will greatly help standardize the process. As Xpert assay provides both TB and Rifampicin resistance results, it is important to weigh the benefit of early detection of drug resistance against the concern that large increases in DR-TB detection would overwhelm advanced diagnostic facilities and nascent drug resistant TB treatment programs. Focusing the Xpert assay testing solely on DR-TB suspects is unlikely to produce large yields as the numbers of failures and retreatment cases at a district level is quite small in most settings. Furthermore, the majority of DR-TB cases will be found among new cases, requiring testing of people with suspected TB rather than patients already receiving TB treatment if meaningful MDR-TB treatment scales up is to take place and finally, the cost plays a vital role, Xpert machine and Cartridge cost huge amount of expenditure to the provider as well as it also increases diagnostic cost and impedes affordability for the patients. Therefore, increasing National tuberculosis control budget and international funding as well as concessional delivery of Xpert machine and cartridge kits is essential in low income countries like Nepal for its effective implementation.

## CONCLUSION

The Xpert assay is a helpful tool in early and accurate diagnosis of PTB and able to give positive results among smear negative samples too. It shows a vital character in the detection of MDR-TB for early treatment of disease as drug sensitivity testing takes longer than a month to give MTB drugs sensitivity patterns. Implementation of Xpert assay in all National DOTS and TB control centers is recommended.

## Figures and Tables

**Fig (1):**
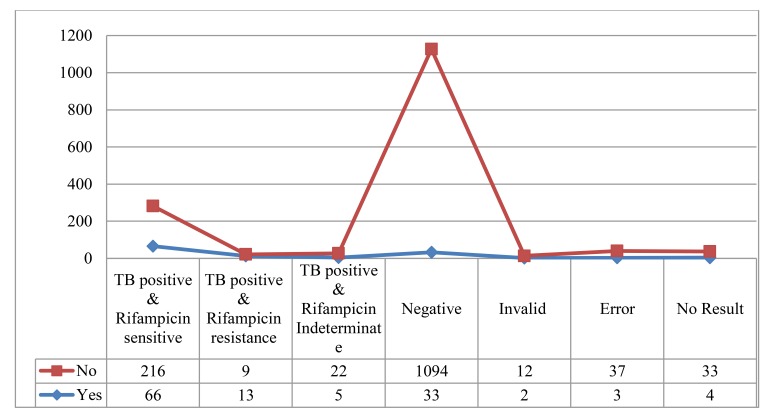
Distribution of Xpert assay result among MDR suspected and MDR not suspected cases

**Table 1:** Distribution of suspected cases and Xpert assay result in different gender.

Gender	Total	TB positive				Negative		
		Rifampicin sensitivity	Rifampicin resistance	Rifampicin Indeterminate	Total		Failed	p-value
Male	992	205	18	20	243	692	57	<0.005
Female	557	77	4	7	88	425	34	
Total	1549	282	22	27	331	1127	91	

**Table 2:** Distribution of suspected cases and Xpert assay result in different age groups.

Age of patients	Total	TB positive						p-value
		Rifampicin Sensitivity	Rifampicin Resistance	Rifampicin Indeterminate	Total	Negative	Failed	
0-20	164	34	5	1	40	116	8	0.002
21-40	495	102	13	13	128	339	28	
41-60	503	84	4	8	96	361	26	
61-80	356	58	0	4	62	267	27	
81-100	31	4	0	1	5	24	2	
Total	1549	282	22	27	331	1127	91	

**Table 3:** Distribution of suspected cases and Xpert assay result, according to treatment history.

Clinical history	Total	TB positive				Negative	failed	p-value
		Rifampicin sensitivity	Rifampicin resistance	Rifampicin Indeterminate	Total			
New suspects	1455	234	10	24	268	1103	84	0.000
Previously treated	94	48	12	3	63	24	7	
Total	1549	282	22	27	331	1127	91	

**Table 4:** Distribution of suspected cases and Xpert assay result, according to the HIV status.

HIV Status	Total		TB positive				Negative	Failed
	Male	Female	Rifampicin sensitivity	Rifampicin resistance	Rifampicin Indeterminate	Total		
HIV positive	8	1	2	0	0	2	6	1
HIV negative	984	556	280	22	27	329	1121	90
Total	992	557	282	22	27	331	1127	91

**Table 5:** Distribution of suspected cases and Xpert assay result among AFB smear result.

	AFB result	TB Positive by Xpert assay				Negative by Xpert assay	Failed	Total
		Rifampicin sensitivity	Rifampicin Resistance	Rifampicin Indeterminate	Total			
Positive	88	57	12	1	70	12	6	88
Negative	1441	222	10	26	258	1099	84	1441
Direct Xpert	-	3	0	0	3	16	1	20
Total	1529	282	22	27	331	1127	91	1549

**Table 6:** Sensitivity and specificity of Xpert assay compared to AFB Results.

		AFB smear Results		Total
		AFB Positive	AFB Negative	
Xpert assay result	MTB detected	70	258	328	PPV =21.3%
	MTB not detected	12	1099	1111	NPV =98.9%
Total		82	1357	1439
		Sensitivity = 85.4%	Specificity= 81%

Note: - PPV: Positive Predictive Value, NPV: Negative Predictive Value
